# Editorial: Therapeutic Effects of Herbal Medicines: How Can We Best Investigate Bioactive Metabolites?

**DOI:** 10.3389/fphar.2022.878789

**Published:** 2022-03-29

**Authors:** Shuai Ji, Wenzhi Yang, Bin Chen, Xionghao Lin, Wei Song, Marcello Locatelli

**Affiliations:** ^1^ Department of Pharmaceutical Analysis, Xuzhou Medical University, Xuzhou, China; ^2^ Tianjin State Key Laboratory of Modern Chinese Medicine, Tianjin University of Traditional Chinese Medicine, Tianjin, China; ^3^ Affiliated Hospital of Integrated Traditional Chinese and Western Medicine, Nanjing University of Chinese Medicine, Nanjing, China; ^4^ College of Medicine, Howard University, Washington, DC, United States; ^5^ Peking Union Medical College Hospital (CAMS), Beijing, China; ^6^ University of Studies G. d’Annunzio Chieti and Pescara, Chieti, Italy

**Keywords:** herbal medicines, pharmacodynamics, pharmacological effects, pharmacokinetics, bioactive compounds, bioactive metabolites

Since ancient times, natural products have always been used as remedies for more or less serious pathologies. The great advantage of traditional medicine lies above all in the wealth of experience obtained “in the field” by experimenting with different natural products and different preparations to deal with specific diseases.

Only recently, starting from the information of traditional medicine, an attempt has been made to apply a more “scientific” approach, trying to actually evaluate which molecules present in the natural preparation have the therapeutic effect.

The awareness of being able to “take a cue” from the natural world in the process of developing new drugs has also developed from this approach, especially as these compounds are generally well tolerated and with reduced (or no) side effects.

In this context, therefore, traditional medicine plays a predominant role in the discovery of new drugs based on natural products, leading to the continuous need to study new herbal matrices for pharmaceutical and nutraceutical purposes, coupled with a continuous progress of the techniques applied to the characterization of natural matrices and to the evaluation of the observed biological activities, in order to better identify the bioactive compounds.

Herbal medicines contain hundreds or even thousands of primary and secondary metabolites, and it is a vital task for pharmacologists to explore which components contribute to the therapeutic effects of herbal medicines and which compounds do not. The complexity and low content of the chemical constituents of these metabolites in herbal medicines pose complex challenges. Up to now, the active components of most herbal medicines remain obscure, which hinders further pharmacological study and development of herbal medicines. In this scenario, the possibility of evaluating and characterizing herbal medicines is of great importance in order to obtain a product that is safe for human health, standardized, whose effects have been studied and evaluated from all points of view.

In general, the absorption of these metabolites needs to be understood in order to evaluate their potential therapeutic effects. Up to now, pharmacologists have tried many methods and techniques to explore the pharmacodynamics of herbal medicines. This includes the *in vivo* characterization of metabolites by pharmaco-metabonomics techniques or *ex vivo* models focusing on the delivery, for example, in the gastrointestinal tract.

In this Research Topic, the main goal aims to attract innovative original contributions in the interdisciplinary area in order to understand the relative impact of different compounds/compound classes to reported pharmacological effects, but also to highlight the state of the art on profiling of metabolites’ pharmacokinetics *in vivo*, on new unreported biological activities or biological targets, and on new bioactive compounds as leads for the pharmaceutical industry.

This result can be achieved through a multidisciplinary approach that involves not only pharmacology and botany, but also disciplines such as analytical chemistry (which guarantees the quality and reproducibility of data), pharmaceutical chemistry, physiology, and biochemistry.

Through an integration of these disciplines and knowledge, it is possible to describe and characterize most of the observed.

In the papers accepted after peer review in this Research Topic, it is also highlighted that in recent years the search for products of natural origin that can be used as they are or as lead compounds for the pharmaceutical development of new drugs is increasingly a central element of scientific research.

What we have seen so far has foundations in the traditional use of many products of plant origin, as highlighted by Zeng et al., Fan et al., and Wang et al. Today these uses are being rediscovered, however, trying to follow a more scientific and systematic approach, associating what has been traditionally observed with chemical characterization and innovative techniques for evaluating biological activity in order to find a possible correlation. Han et al., Wan et al., Zheng Li et al., and Xu et al., which report approaches to proteomics, metabolomics and new strategies, respectively, amply highlight this.

Other authors such as He et al., Gao et al., and Yang et al., focus their attention on specific markers of some native products and try to evaluate their targets and their respective biological effects.

Jing et al., Gao et al., Hui-Yu Li et al., and Zixuan Li et al., instead closely evaluate traditional uses with respect to specific pathways involved in various pathologies.

Nan et al., Xuerui Wang et al., and Su et al., instead evaluate how pills and capsules containing natural products can show therapeutic and/or preventive effects against human pathologies. Interesting the work of Pan et al., which reports how metabolomics studies carried out by hyphenated analytical approaches of LC-MS allow to evaluate and correlate the effects of EGCG on A549 cells.

